# On the Evils of Consanguinity in Marriage

**Published:** 1856-09

**Authors:** 

**Affiliations:** Geneva


*On the evils of Consanguinity in Marriage.* By Dr. RILLIET of Geneva.

A letter from the author, containing a statement of his researches in reference to the influence exercised by consanguinity upon the offspring of marriage, was read before the Académie de Médicine at their sitting of the 13th of May. The substance of the letter was: that at Geneva a considerable number of marriages take place between relatives; that attention has during many years been attracted to the unhappy consequences resulting from this circumstance, and affecting the health and even the lives of the children. These consequences are, first, absence of conception; 2nd, retardation of conception; 3rd, imperfect conception, (miscarriage;) 4th, imperfect offspring, (monstrosities;) 5th, offspring more specially liable to diseases of the nervous system, and in order of frequency: epilepsy, imbecility or idiocy, deaf-mutism, paralysis, various cerebral diseases; 6th, a lymphatic offspring predisposed to the diseases which spring from the scrofulo-tuberculous diathesis; 7th, an offspring which dies at an early age, in a larger proportion than children born under other circumstances; 8th, an offspring which, if it passes the first period of infancy, is less capable than others of resisting disease and death. To these rules there are exceptions, due either to the state of health of the progenitors, or to the dynamic conditions in which the parents happen to be at the time of connexion. Thus: 1, it is seldom that all the children escape the evil influence; 2, in the same family some are affected, others are spared; 3, those who are affected are almost never similarly circumstanced in the same family—that is to say, one is epileptic, while another is a deaf mute, &c.—*Dublin Med. Press from Gazette Medicale.*

